# 

**Published:** 1904-12

**Authors:** 


### The Library of the Bristol Medico-Chirurgical Society.

*The following donations have been received since the publication of the List in September:*

*November 30th, 1904.*

| | |
|---|---|
| Association of Medical Librarians (1) ... ... ... | 5 volumes. |
| Medical Officer of Health of Bristol (2)... ... ... | 1 volume. |
| F. H. Edgeworth, M.B. (3) ... ... ... ... ... | 30 volumes. |
| L. M. Griffiths (4) ... ... ... ... ... ... ... | 12 ,, |
| Council of the Royal College of Physicians of London (5) ... ... ... ... ... ... ... | 1 volume. |
| R. Shingleton Smith, M.D. (6)... ... ... ... ... | 4 volumes. |
| The Surgeon-General, United States Army (7) ... | 1 volume. |
| Council of University College, Bristol (8) ... ... | 1 ,, |

### FIFTY-FOURTH LIST OF BOOKS.

The titles of books mentioned in previous lists are not repeated.

The figures in brackets refer to the figures after the names of the donors, and show by whom the volumes were presented. The books to which no such figures are attached have either been bought from the Library Fund or received through the *Journal.*

*Atlas of Illustrations of Clinical Medicine, Surgery, and Pathology, An* Fasc. X.—XII. 1904

**Bartholow, R.** ... *Medical Electricity* ... ... ... ... ... ... (3) 3rd Ed. 1897

**Beale, L. S.** ... *On Slight Ailments* ... ... ... ... ... ... ... ... (3) 1880

**Black, C. B.** ... *Guide to South of France, &c.* ... ... (3) 3rd Ed. [N.D.]

**Bryson, A.** ... ... *Epidemic Fevers of Sierra Leone* ... ... ... ... (3) 1849

**Carter, R. B.** ... *Eyesight: Good and Bad* ... ... ... ... ... ... (3) 1880

*Catalogue (Index-) of the Library of the Surgeon-General's Office, United States Army* ... ... ...2nd Ser., Vol. IX. (7) 1904

*Catalogue of Accessions to the Library of the Royal College of Physicians during the Year ending July, 1904* ... ... (5) 1904

**Coles, A. C.** ... *Clinical Diagnostic Bacteriology* ... ... ... ... ... 1904

**Cooley, A. J.** ... *Cyclopædia of Practical Receipts.* 2 vols. (3) 6th Ed. 1880

**Cowen, R. J.** .. *X-rays: their Employment in Cancer and other Diseases* 1904

**Dobell, H.** ... ... *On Winter Cough* ... ... ... ... ... ... (3) 3rd Ed. 1875

,, ... ... *The Mont Dore Cure* ... ... ... ... ... ... ... (3) 1881

**Edwards, G. D.**... *Notes on Assouan* ... ... ... ... ... ... ... ... ... 1904

**Fothergill, J. M.** *Digitalis* .. ... ... ... ... ... ... ... ... ... (3) 1871

**Gairdner, W.** ... *Gout* ... ... ... ... ... ... ... ... ... (3) 4th Ed. 1860

**Gibson, G. A.** ... *Nervous Affections of the Heart* ... ... ... ... ... 1904

**Griffith and A. Henfrey, J. W.** *Micrographic Dictionary* ... (3) 3rd Ed. 1875

**Haig, A.** ... ... *Uric Acid* ... ... ... ... ... ... ... ... (3) 2nd Ed. 1874

**Harrison, R.** ... *The Ambulance in Civil Life* ... ... ... [6th Ed.] 1904

**Hassall, A. H.** ... *Inhalation Treatment* ... ... ... ... ... ... (3) 1885

**Henfrey, J. W. Griffith and A.** *Micrographic Dictionary* ... (3) 3rd Ed. 1875

**Hewer, Mrs. J. L.** *Our Baby* ... ... ... ... ... ... ... ... ... 9th Ed. 1904

**Hooper, R.** ... *Physician's Vade-Mecum* ... ... ... ... (3) New Ed. 1812

**James, P.** ... ... *Therapeutics of Respiratory Passages* ... ... ... (3) 1885

**Johnson, G.** ... *Lectures on Bright's Disease* ... ... ... ... ... (3) 1873

**Jones, H. L.** ... *Medical Electricity* ... ... ... ... ... ... ... 4th Ed. 1904

**Kaminer, H. Senator and S.** [Eds.] *Health and Disease in Relation to Marriage.* (Tr. by J. Dulberg.) Vol. I. ... ... 1904

**Lake, R.** ... ... *Diseases of the Ear* ... ... .. ... ... ... 2nd Ed. 1904

**Lamb, W.** ... ... *Examination of Throat, Nose, and Ear* ... ... ... 1904

**Lawrence, W.** ... *Lectures on Man* ... ... ... ... ... (4) 9th Ed. 1844

**Lee, E.** ... ... *Baths of Germany* ... ... ... ... ... (4) 4th Ed. 1863

**Leedham-Green, C.** *On the Sterilisation of the Hands* ... ... ... ... ... 1904

*Life Assurance at the Commencement of the Nineteenth Century* ... ... (6) 1901

**Love, J. K.** ... ... *Diseases of the Ear* ... ... ... ... ... ... ... ... 1904

**Mackenzie, M.** ... *Hay Fever*... ... ... ... ... ... ... (3) 5th Ed. 1887

**Moullin, C. M.** ... *Enlargement of the Prostate* ... ... ... ... 3rd Ed. 1904

**Muirhead, C.** ... *Causes of Death in Scottish Widows' Fund from 1874 to 1894* ... ... ... ... ... ... ... ... (6) 1902

**Mummery, P. L.** *After-treatment of Operations* ... ... ... ... 2nd Ed. 1904

**Poincaré [—]** ... *Leçons sur la Physiologie du Système nerveaux périphérique* ... ... ... ... ... ... ... ... (4) 1876

**Prichard, A.** ... *Ten Years of Operative Surgery in the Provinces.* 2 parts (3) 1862-63

**Rawling, L. B.** ... *Landmarks and Surface Markings* ... ... ... ... ... 1904

**Richardson, B.W.** *Diseases of Modern Life* ... ... ... ... (3) 3rd Ed. 1876
**Santi, P. R. W. De** *Malignant Disease of the Larynx* ... ... ... ... ... ... 1904
**Senator and S. Kaminer, H.** [Eds.] *Health and Disease in Relation to Marriage.* (Tr. by J. Dulberg.) Vol. I. ... ... 1904
**Smith, W. J.** ... *Shipmasters' Medical and Surgical Help* .. ... ... (3) 1895
**Steavenson, W. E.** *Electrolysis in Surgery* ... ... ... ... ... ... ... (3) 1890
**Taylor, J.** ... ... *Bristol and Clifton: Old and New*... ... ... ... (4) [1877]
**Thompson, Sir H.** *Diseases of the Prostate* ... ... ... ... (3) 5th Ed. 1883
,, *Surgery of the Urinary Organs* ... ... ... ... (3) 1884
**Treves, Sir F.** ... *Student's Handbook of Surgical Operations.* New Ed. (Revised by the author and J. Hutchinson, jun.) ... ... .. ... ... ... ... ... ... ... ... 1904
**Vincent, R.** ... *The Nutrition of the Infant* ... ... ... ... 2nd Ed. 1904
**Walker, Jane H.** *The Modern Nursing of Consumption* ... ... ... ... [1904]
**Walker, N.** ... *An Introduction to Dermatology* ... ... ... 3rd Ed. 1904
**Walsham, W. J.** . *Handbook of Surgical Pathology.* 3rd Ed. (Ed. by H. J. Paterson) ... ... ... ... ... ... ... ... 1904
**Wheeler, A.** ... *Medicine and Therapeutics* ... ... ... ... ... (3) 1894
**Williams, W.** ... *Deaths in Childbed* ... ... ... ... ... ... ... ... ... 1904
**Woakes, E.** ... *Post-Nasal Catarrh* ... ... ... ... ... (3) 3rd Ed. 1884

### TRANSACTIONS, REPORTS, JOURNALS, &c.

American Journal of Medical Sciences, The ... ... Vol. CXXVII. 1904
American Medicine ... ... ... ... ... ... ... ... ... ... ... Vol. VII. 1904
Boston Medical and Surgical Journal, The ... ... ... Vol. CXL. 1904
Bristol and Clifton Directory, Mathew's ... ... ... ... ... (4) 1865-69
Bristol Directory, Hunt's ... ... ... ... ... ... ... ... ... ... (4) 1850
Bristol Health Report for 1903 ... ... ... ... ... ... ... ... (2) 1904
British Medical Journal, The ... ... ... ... ... ... ... ... Vol. I. for 1904
Calendar of University College, Bristol, for 1904-05 ... ... ... (8) 1904
Cincinnati Lancet-Clinic, The ... ... ... ... ... ... ... Vol, XCI. 1904
Clinical Society's Transactions ... ... ... ... ... Vol. XXXVII. 1904
Congrès (xiv^e) international de Médecine, Madrid—Comptes rendus Section de Thérapeutique; Volume général ... ... ... ... (6) 1904
Finska Läkaresallskapets nittonde allmänna möte i Helsingfors, 1903, Forhandlingar vid ... ... ... ... ... ... ... ... ... ... ... ... 1904
Glasgow Medical Journal, The ... ... ... ... ... ... Vol. LXI. 1904
International Clinics ... (1) 2nd Series, Vols. III., IV., 1892-93; 3rd Series, Vol. II., 1893; 7th Series, Vol. IV., 1898; 13th Series, Vol. I. 1903
Journal of Medical Research, The ... ... ... ... ... ... Vol. XI. 1904
Journal of Obstetrics and Gynæcology of the British Empire, The Vol. V. 1904
Journal of the American Medical Association, The Vols. XLI., XLII. 1903-04
Lancet, The ... ... ... ... ... ... ... ... ... ... ... ... Vol. I. for 1904
Library World, The ... ... ... ... ... ... ... ... ... ... ... Vol. VI. 1904
Liverpool Medico-Chirurgical Journal, The ... ... ... Vol. XXIII. 1903
Louisville Monthly Journal, The ... ... ... .. ... ... Vol. X. 1903-04
Medical News, The ... ... ... .. ... ... ... ... ... Vol. LXXXIV. 1904
Medical Press and Circular, The ... ... ... ... ... Vol, CXXVIII. 1904

| | | |
|---|---|---|
| Medico-Chirurgical Transactions ... ... ... ... | [Vol. LXXXVII.] | 1904 |
| Münchener medizinische Wochenschrift ... ... ... ... | Hälfte I. for | 1904 |
| New York, Transactions of the Medical Society of the State of ... ... | | 1904 |
| Ohio State Medical Society, Transactions of the ... ... ... ... ... | | 1904 |
| Physical Deterioration Committee, Report of the. | 3 vols. ... ... ... | 1904 |
| Practitioner, The ... ... ... ... ... ... ... ... ... | [Vol. LXXII.] | 1904 |
| Progressive Medicine ... ... ... ... ... ... | Vols. I., 1903; III. | 1904 |
| Registrar-General's Quarterly Returns of Marriages, Births, and Deaths in England. 2 vols. ... ... ... ... ... ... ... ... ... ... | (4) | 1878–81 |
| Rockefeller Institute for Medical Research, Studies from the... | Vol. II. | 1904 |
| Royal Academy of Medicine in Ireland, Transactions of the | Vol. XXII. | 1904 |
| Sanitary Record, The ... ... ... ... ... ... ... | Vol. XXXIII. | 1904 |
| Scottish Medical and Surgical Journal, The ... ... ... | Vol. XIV. | 1904 |
| South African Medical Record, The ... ... ... ... ... ... | Vol. I. | 1903 |

The Bristol Medical Library keeps an album for newspaper cuttings and other local items relating to medicine, and the Honorary Librarian will always be glad to receive any additions to it.

### MEETINGS OF SOCIETIES.

### Bristol Medico-Chirurgical Society.

### Local Medical Notes.

**University College, Bristol.**—The following appointments have recently been made: F. H. Edgeworth, M.B., B.C., B.A.Cantab., D.Sc.Lond., Joint-Professor of Medicine, *vice* J. E. Shaw, M.B., C.M., resigned. E. W. Hey Groves, M.D., B.S., B.Sc. (Lond.), and W. S. V. Stock, M.D., B.S. Lond., Demonstrators of Anatomy, *vice* J. O. Symes, M.D. Lond., and T. Carwardine, M.B. Lond., resigned. Students of the College have been successful in the following examinations: University of London.—*M.D.:* Carey F. Coombs, H. S. Jenkins. *B.S.* (*Honours*): E. W. Hey Groves, M.D., B.Sc. *M.B.*, *B.S.*, *Group I.:* A. Coleridge. Conjoint Board of the Royal Colleges of Physicians and Surgeons of England.—*First Examination: Biology*—R. C. Clarke, S. H. Kingston. *Practical Pharmacy*—H. W. Goodden, A. J. O. Wigmore. *Second Examination: Anatomy and Physiology*—P. S. Tomlinson. *Final Examination: Surgery*—T. S. Davies,* L. N. Morris, S. C. Hayman.* *Midwifery*—H. O. Gough, A. N. Thomas, M. R. O. Wilson. L.D.S. Eng.—*Final Examination, Part I.*—W. J. Jones; *Part II.*—A. J. Mundy.*

**Winsley Sanatorium.**—Dr. Salmond has been appointed Resident Medical Officer, and Miss Jessie Creighton as Matron, and have entered on their duties.

**Openairia at the Zoological Gardens, Clifton.**—The report of the Secretary to the Committee of the fête has just been received, showing a balance in favour of the Winsley Sanatorium for Consumptives amounting to £1,529 17s. 2d.

**Public Health, Bristol.**—For these notes we are much indebted to the Medical Officer of Health, Dr. D. S. Davies.

The general death-rate for the third quarter of 1904, 13.3, compares favourably with the average rate for the seventy-six largest English towns, 17.5, and is the lowest rate recorded in those towns having a population over 200,000.

*Extension of City Boundaries.*—On October 1st the city was extended by the addition of Horfield, Westbury-on-Trym, Shirehampton, and part of Henbury. The effect of these extensions

* Denotes qualification.

is to increase the area of the city, which in 1897 was only 4,661 acres, to 17,000 acres, and the population to 354,443. The city is now the sixth in size by population, and the fourth in acreage, of the provincial cities.

*Hospitals.*—The deficiency in hospital accommodation which has long characterised Bristol is being surely if slowly made good, but the city is still alone amongst the largest cities in not even approaching the required minimum of one bed per 1,000 inhabitants for isolation purposes. The adequate number of beds for the city would be 350. At Ham Green the new block will provide isolation room (at 2,000 cubic feet per bed) for 58 beds, and these added to the 76 previously built gives 134 beds at full cubic space. In times of pressure 170 beds can be made up in this hospital by somewhat curtailing the cubic space allowance. With the aid of the Clift House (temporary) accommodation, and of that at Nover's Hill (originally provided for small-pox), the available beds in the city number about 240, with some crowding.

*Small-pox* has continued to make its appearance from time to time, chiefly attracted by the dock works at Avonmouth. The type of disease introduced into Bristol is still of an extremely modified kind, and in many cases extraordinarily mild, increasing the chances of "missed" cases. By carefully following up clues, and by persistent search for any missed case, success in prevention has been uniform, as for the past ten years.

The recent conference on the spread of disease by vagrants, held on 10th November at the County Hall, Spring Gardens, London, was attended by a large number of delegates from the county councils, county and Metropolitan boroughs, and from the Metropolitan Asylums Board. Eighteen resolutions were discussed and adopted, comprising general measures for the prevention of the spread of infectious disease by vagrants, common lodging houses and casual wards, labour bureaux, labour colonies, children of vagrants; and a committee was appointed to give practical effect to the resolutions by such constitutional methods as may best enlist the sympathy and co-operation of the Government. The Medical Officer of Health for Bristol was appointed one of the Members of Committee of the County Borough Councils.

The conference disclosed the fact that, in the majority of towns, the vagrant is much dreaded as an efficient agent in the spread of small-pox, and common lodging houses seem to act as frequent centres of infection. Curiously enough, in Bristol, though tramps not infrequently introduce small-pox, they have not for ten years past proved efficient agents for promoting an epidemic, nor have the common lodging houses responded to the introductions.

Our experience has been that private workers visiting friends have started the only two substantial outbreaks of recent years, but in no case have more than fifteen cases been permitted to exist at any one time for the past ten years. Direct personal care and minute attention to "missed" cases and to detail appear in general to be sufficient to meet, with the aid of revaccination, the insistent but straightforward attacks of small-pox.

*Diphtheria* is a disease of quite another kind, but it is satisfactory to note that the fatality of the disease, which has given so much trouble since 1900, is continuously declining, as shown by these figures:—

DIPHTHERIA MORTALITY IN BRISTOL.

Deaths per 100,000 persons living.

| | |
|---|---|
| 1899 ... ... ... ... | 10 |
| 1900 ... ... ... ... | 31 |
| 1901 ... ... ... ... | 37 |
| 1902 ... ... ... ... | 55 |
| 1903 ... ... ... ... | 35 |
| 1904—1st quarter ... ... | 37 |
| 2nd ,, ... ... | 32 |
| 3rd ,, ... ... | 17 |

*Enteric Fever.*—This disease is by no means prevalent in Bristol, though the city shared in the usual increase during the third quarter of the year. This year seventy-eight cases were notified in that period, a number in excess of the phenomenally low (and wet) year 1903, when forty-six only were returned, but well within the average of the past five years.

*Diarrhœa.*—The third quarter of the year is characterised, especially in a hot season, by considerable infantile diarrhœa mortality. Although Bristol shares the autumnal rise in mortality with the other towns, the diarrhœa rate (1.61) is the lowest of all towns having a population over 200,000. This is only notable because considerable excitement was caused in August by an unfounded newspaper article, copied widely into the London newspapers, stating that an epidemic of enteritis had been caused among the children of the poorer districts in Bristol by the use of an injurious preservative in milk: there was no foundation of any sort for this report, which seems to have arisen from inexperience and misconception.

### INDEX.

(R.) = Review.

Abdominal tuberculosis, Vagaries of—Dr. J. Swain, 31.
Adenoids—Dr. W. Wingrave (R.), 359.
Allchin, Dr. W. H. [Ed.]—A manual of medicine (R.), 147.
Alopecia areata, 344.
Ambulance, A manual of—Dr. J. S. Riddell (R.), 267.
American Dermatological Association, Transactions of the (R.), 166.
American Pediatric Society, Transactions of the (R.), 173.
American Physicians, Transactions of the Association of (R.), 263.
American Surgical Association, Transactions of the (R.), 159.
Andrewes, Dr. F. W.—Lessons in disinfection and sterilisation (R.), 170.
Aneurysm of the ascending aorta—Dr. F. H. Edgeworth, 23.
Ankylostomiasis, 134.
Armies in the field, Prevention of disease in—R. Caldwell (R.), 261.
Asthma in relation to the nose—Dr. A. Francis (R.), 156.
Ateleiosis, 249.

Bacteriological technique—Dr. J. W. H. Eyre (R.), 69.
Bacteriology, Clinical diagnostic—Dr. A. C. Coles (R.), 350.
Bandaging and dressings, Surgical—W. J. Smith (R.), 72.
Bastian, Dr. H. C.—Studies in heterogenesis (R.), 145.
Beale, Dr. P. T. B.—Aids to physiology (R.), 69.
Beddoe, Dr. J.—The Long Fox lecture, 303.
Benham, Obituary notice of Dr. Harry Arthur, 286.
Bennett, Sir W.—Modern nursing in private practice (R.), 167.
Biographic clinics—Dr. G. M. Gould (R.), 151.
Blackwater fever—Dr. E. C. Williams, 128.
Bright's disease, Surgical treatment of, 253.
Bright's disease treated by renal decapsulation, 62.
Bristol, Health of—Dr. D. S. Davies, 55, 382.
Bristol Medico-Chirurgical Society, 87, 188, 377.
Bristol Royal Infirmary, 175.
British journal of children's diseases, The (R.), 166.
British Medical Association, Oxford meeting of the, 268.

Cæsarean section, Case of—Dr. W. C. Swayne, 120.
Caldwell, Dr. W. A. Pusey and E. W.—The practical application of the Röntgen rays in therapeutics and diagnosis (R.), 71.
Caldwell, R.—The prevention of disease in armies in the field (R.), 261.
Campbell, Dr. K.—The refraction of the eye (R.), 71.
Cancer and pre-cancerous changes—G. H. Fink (R.), 168.
Cancer of the uterus—Dr. A. H. N. Lewers (R.), 265.
Carter and A. H. Cheatle, R. B.—Sight and hearing in childhood (R.), 170.
Carter, R. B.—Doctors and their work (R.), 258.
Carwardine, T.—Report on surgery, [253.
Cerebro-spinal fluid, Cyto-diagnosis of, 335.
Charities, The misuse of medical—Miss E. Sturge, 273.
Cheatle, R. B. Carter and A. H.—Sight and hearing in childhood (R.), 170.
Cheyne and F. F. Burghard, Drs. W. W.—A manual of surgical treatment (R.), 70.

Childhood, Sight and hearing in—R. B. Carter and A. H. Cheatle (R.), 170.
Children, The care and feeding of—Dr. L. E. Holt (R.), 356.
Chorea and rheumatism, The relationship of—Dr. J. J. S. Lucas, 239.
Clarke, Dr. J. M.—Report on medicine, 334.
Clarke, Dr. W. B.—The meaning of a modern hospital (R.), 353.
Cleft palate and hare-lip.—Dr. E. Owen (R.), 355.
Clemow, Dr. F.—The geography of disease (R.), 69.
Clinical methods—Dr. C. H. Melland (R.), 156.
Coats, Dr. J.—A manual of pathology (R.), 167.
Cocaine, General anæsthesia by intra-spinal injections of—Dr. A. S. Gubb, 231.
Cocainisation, Surgical analgesia by spinal—W. J. Greer, 235.
Coles, Dr. A. C.—Clinical diagnostic bacteriology (R.), 350.
Columbia University, Studies from the department of pathology of the College of Physicians and Surgeons (R.), 169.
Consumption, Handbook of open-air treatment—Drs. C. Reinhardt and D. Thomson (R.), 172.
Consumption, Prevention of, 94; —Dr. A. Hillier (R.), 171.
Consumption, The sanatorium treatment of—Dr. T. N. Kelynack (R.), 154.
Crossman, Edward, In memoriam, 193.
Cyto-diagnosis, 335.

Davies, Dr. D. S.—Mortality and sickness in the city of Bristol for the year 1903, 55.
Deciduoma malignum, 140.
Dermatological Association, Transactions of the American (R.), 166
Dermatology, Report on—Dr. W. K. Wills, 342.
Disease, Geography of—Dr. F. Clemow (R.), 69.
Disinfection, Lessons in—Dr. F. W. Andrewes (R.), 170.
Dispensing made easy—Dr. W. G. Sutherland (R.), 163.
Doctors and their work—R. B. Carter (R.), 258.
Dowse, Dr. T. S.—Lectures on massage and electricity in the treatment of disease (R.), 267; The pocket therapist (R.), 167.
Drugs—H. W. Gadd (R.), 162.

Eager, Dr. R.—Problems in psychology, 289.
Ear, Diseases of the—R. Lake (R.), 150.
Eccles, W. McA.—The imperfectly descended testis (R.), 160.
Edgeworth, Dr. F. H.—Aneurysm of the ascending aorta, 23.
Edinburgh medical journal, The (R.), 171.
Edinburgh Obstetrical Society, Transactions of the (R.), 164.
Editorial notes, 74, 175, 268, 362.
Electrical department, Some cases from an—Dr. G. Parker, 112.
Electricity, Lectures on massage and—Dr. T. S. Dowse (R.), 267.
Electro-therapy, Polyphase currents in—Dr. G. Herschell (R.), 173.
Ellison, M.A.—A manual for students of massage (R.), 360.
Epidemiological Society of London, Transactions of the (R.), 264.
Erythema infectiosum, 345.
Ethyl chloride—A. L. Flemming, 228; C. H. Whiteford, 49.
Exostosis, Subungual—W. R. Williams, 17.
Eye, Diseases of the—G. Lawson (R.), 266; Dr. H. R. Swanzy (R.), 169.
Eye symptoms as aids in diagnosis—Dr. E. Magennis (R.), 266.
Eyre, Dr. J. W. H.—The elements of bacteriological technique (R.), 69.

Fink, G. H.—Cancer and precancerous changes: their origin and treatment (R.), 168.
"First aid"—Drs. F. J. Warwick and A. C. Tunstall (R.), 171.
Firth, Dr. J. L.—On torsion of the spermatic cord, 320; Report on surgery, 62.
Fisher, Dr. T.—Hæmatemesis associated with small white kidneys, 243; Report on medicine, 58.
Fisher and N. Neild, Drs. T.—A case of congenital hypertrophic stenosis of the pylorus, 123.

Flemming, A. L.—Ethyl chloride: a few practical remarks, 228.
Football injuries—R. H. A. Whitelocke (R.), 161.
Fortescue-Brickdale, Dr. J. M.—Lac vinum infantum: a review of the work of infant milk depôts, 197; Note on the condition of the blood corpuscles in blackwater fever, 130.
Fothergill, Dr. W. E.—Manual of midwifery (R.), 164.
Fox lecture, The Long, 303, 362.
Francis, Dr. A.—Asthma in relation to the nose (R.), 156.

Gadd, H. W.—Drugs: their production, preparation, and properties (R.), 162.
Gall-bladder and bile-ducts, Diseases of the—A. W. M. Robson (R.), 159.
Gould, A. P.—Elements of surgical diagnosis (R.), 158.
Gould, Dr. G. M.—Biographic clinics (R.), 151.
Greer, W. J.—On surgical analgesia by spinal cocainisation, 235.
Groves, Dr. E. W. H.—A case of tubal gestation producing severe hemorrhage without rupture, 46.
Gubb, Dr. A. S.—The induction of general anæsthesia by intra-spinal injections of cocaine, 231.
Guy's hospital reports (R.), 155.

Hæmatemesis associated with small white kidneys—Dr. T. Fisher, 243.
Haines, Drs. F. Peterson and W. S.—A textbook of legal medicine and toxicology (R.), 257.
Hamer, Dr. W. H.—Manual of hygiene (R.), 348.
Hands, On the sterilisation of the—Dr. C. Leedham-Green (R.), 351.
Hare-lip, Cleft palate and—Dr. E. Owen (R.), 355.
Haultain, Dr. F. W. N.—A practical handbook of midwifery (R.), 169.
Heine, Dr. B.—Operationen am ohr (R.), 262.
Herman, Dr. G. E.—Diseases of women (R.), 259.
Herring, Dr. H. T.—The sterilisation of urethral instruments (R.), 160.
Herschell, Dr. G.—Polyphase currents in electro-therapy (R.), 173.
Heterogenesis, Studies in—Dr. H. C. Bastian (R.), 145.
Hewlett, Dr. R. T.—Serum therapy (R.), 167.
Hillier, Dr. A.—The prevention of consumption (R.), 171.
Hip-joint, Congenital dislocation of the, 255.
Hip-joint, Tuberculous disease of the—C. A. Morton, 207.
Histology, Atlas and epitome of human—Dr. J. Sobotta (R.), 68.
Holt, Dr. L. E.—The care and feeding of children (R.), 356.
Homfray, C. T. Kingzett and D.—A pocket dictionary of hygiene (R.), 174.
Hospital administration, 272, 273.
Hospital, The meaning of a modern—Dr. W. B. Clarke (R.), 353.
Hygiene, Manual of—Dr. W. H. Hamer (R.), 348.
Hygiene, Pocket dictionary of—C. T. Kingzett and D. Homfray (R.), 174.

Infantilism, Case of—Dr. E. C. Williams, 247.
Insanity in every-day practice—E. G. Younger (R.), 357.
Insanity, The treatment of, 364.
Instruments, Sterilisation of urethral—H. T. Herring (R.), 160.

Jamaica, Leprosy in—Dr. W. D. Neish and T. J. Tonkin, 1.
Jardine, Dr. R.—Practical textbook of midwifery for nurses (R.), 350.
Johns Hopkins hospital reports (R.), 157.
Joint disease, Some forms of—Dr. W. H. White, 97.
Jones, A. H. Tubby and R.—Modern methods in the surgery of paralyses (R.), 72.

Kaminer, Drs. H. Senator and S. [Eds.]—Health and disease in relation to marriage and the married state (R.), 347.
Kelynack, Dr. T. N.—The sanatorium treatment of consumption (R.), 154.
Kingzett and D. Homfray, C. T.—A pocket dictionary of hygiene (R.), 174.

Lac vinum infantum ; a review of the work of infant milk depôts—Dr. J. M. Fortescue-Brickdale, 197.
Lake, R.—Handbook of diseases of the ear (R.), 150.
Langenhagen, Dr. M. L.—Muco-membranous entero-colitis (R.), 172.
Lawson, G.—Diseases and injuries of the eye (R.), 266.
Leech, Dr. D. J.—The pharmacological action and therapeutic uses of nitrites and allied compounds (R.), 360.
Leedham-Green, Dr. C.—On the sterilisation of the hands (R.), 351.
Legal medicine and toxicology, A textbook of—Drs. F. Peterson and W. S. Haines (R.), 257.
Leprosy in Jamaica—Dr. W. D. Neish and T. J. Tonkin, 1.
Lewers, Dr. A. H. N.—Cancer of the uterus (R.), 265; Diseases of women (R.), 172.
Library of the Bristol Medico-Chirurgical Society, 85, 185, 283, 374.
Lindsay, Dr. J. A.—Clinical lectures on diseases of the lungs and heart (R.), 152.
Local medical notes, 94, 191, 288, 382.
Lucas, Dr. J. J. S.—The relationship of chorea and rheumatism, 239.
Lumbar puncture, 334.
Lungs and heart, Diseases of the—Dr. J. A. Lindsay (R.), 152.
Lupus vulgaris, 342.

Maclagan, Dr. T. J., and his great work, 178.
Macleod, Dr. J. M. H.—Practical handbook of the pathology of the skin (R.), 349.
Maddox, Dr. E. E.—Golden rules of refraction (R.), 170.
Magennis, Dr. E.—Eye symptoms as aids in diagnosis (R.), 266.
Malarial fever, Case of æstivo-autumnal—Dr. J. O. Symes, 126.
Manual treatment of diseases of women—Dr. G. Norström (R.), 265.
Marriage, Health and disease in relation to—Drs. H. Senator and S. Kaminer [Eds.] (R.), 347.
Martindale and Westcott's extra pharmacopœia (R.), 358.
Massage, On—Dr. T. S. Dowse (R.), 267; M. A. Ellison (R.), 360; M. D. Palmer (R.), 170.
Mastication, The physiology of—Dr. J. S. Wallace (R.), 155.
Materia medica and therapeutics—Dr. A. A. Stevens (R.), 73.
Medical defence, 178.
Medical education, 363.
Medicine, A manual of—Dr. W. H. Allchin [Ed.] (R.), 147.
Medicine, Reports on—Dr. J. M. Clarke, 334; Dr. T. Fisher, 58; Dr. G. Parker, 131, 249.
Medico-chirurgical transactions (R.), 361.
Melland, Dr. C. H.—A pocket-book of clinical methods (R.), 156
Mental diseases and degeneracy, The physiognomy of—Dr. J. Shaw (R.), 263.
Mesenteric cyst, Case of—A. W. Prichard, 332.
Mesenteric embolism and thrombosis, 339.
Midwifery for nurses—Dr. R. Jardine (R.), 350.
Midwifery, Manual of—Dr. W. E. Fothergill (R.), 164.
Midwifery, Practical handbook of—Dr. F. W. N. Haultain (R.), 169.
Miles, Drs. A. Thomson and A.—Manual of surgery (R.), 148, 358.
Milk depôts, Review of the work of infant—Dr. J. M. Fortescue-Brickdale, 197
Milk: its production and uses—Dr. E. F. Willoughby, (R.), 153.
Milroy, Drs. J. A. and T. H.—Practical physiological chemistry (R.), 354.
Mineral springs and climates, Therapeutics of—Dr. I. B. Yeo (R.), 162.
Morton, C. A.—Reports on surgery, 136, 255; Some observations on tuberculous disease of the hip-joint in childhood and youth, 207.
Muco-membranous entero-colitis—Dr. M. De Langenhagen (R.), 172.
Mummery, P. L.—The after-treatment of operations (R.), 361.
Myo-fibroma of the uterus, Operation in—Dr. J. Swain, 220

Neild, Drs. T. Fisher and N.—A case of congenital hypertrophic stenosis of the pylorus, 123.
Neish and T. J. Tonkin, Dr. W. D. —Leprosy in Jamaica, 1.
Nephritis, Unilateral chronic, 66.
Nitrites, Action and therapeutic uses of—Dr. D. J. Leech (R.), 360.
Noorden, Dr. C. v.—Diseases of metabolism and nutrition (R.), 352.
Norris, Dr. R. C.—A textbook of obstetrics (R.), 259.
Norström, Dr. G. — The manual treatment of diseases of women (R.), 265.
Nurses, State registration of, 179.
Nursing in private practice, Modern, —Sir W. Bennett (R.), 167.
Nutrition, Diseases of metabolism and—Dr. C. v. Noorden (R.), 352.

Obituary, 193, 286.
Obstetrics, Report on—Dr. W. C. Swayne, 140.
Obstetrics, Textbook of—Dr. R. C. Norris (R.), 259.
Ohr, Operationen am—Dr. B. Heine, (R.), 262.
Operations, The after-treatment of —P. L. Mummery (R.), 361.
Ophthalmic nursing—Dr. S. Stephenson (R.), 170.
Ophthalmological Society of the United Kingdom, Transactions of the (R.), 165.
Overall, Dr. G. W.—A non-surgical treatise on diseases of the prostate gland and adnexa (R.), 264.
Owen, Dr. E.—Cleft palate and hare-lip (R.), 355.

Palmer, M. D.—Lessons on massage (R.), 170.
Pancreas, Surgery of the, 138.
Paralyses, Surgery of—A. H. Tubby and R. Jones (R.), 72.
Parker, Dr. G.—Notes on some cases from an electrical department, 112; Reports on medicine, 131, 249.
Patent medicines, 78.
Pathology, A manual of—Dr. J. Coats (R.), 167.
Pediatric Society, Transactions of the American (R.), 173.
Peterson and W. S. Haines, Drs. F. —A textbook of legal medicine and toxicology (R.), 257.
Pharmacopœia, Martindale and Westcott's extra (R.), 358.
Phthisis, The early diagnosis of, 250; *see* Consumption.
Physiological chemistry, Practical —Drs. J. A. and T. H. Milroy (R.), 354.
Physiology, Aids to—Dr. P. T. B. Beale (R.), 69.
"Practical medicine" (R.), 156.
Prichard, A. W.—Case of mesenteric cyst, 332; Note on the subsequent history of a case of pylorectomy, 53.
Progeria, 249.
Prostate gland, Non-surgical treatise on diseases of the—Dr. G. W. Overall (R.), 264.
Prostatectomy, 136.
Psychology, Problems in—Dr. R. Eager, 289.
Public health, Ministry of, 179.
Purpura, 345.
Purpura, Henoch's, 58.
Pusey and E. W. Caldwell, Dr. W. A.—The practical application of the Röngten rays in therapeutics and diagnosis (R.), 71.
Pylorectomy, The subsequent history of a case of—A. W. Prichard, 53.
Pylorus, Congenital hypertrophic stenosis of the—Drs. T. Fisher and N. Neild, 123.

Radiotherapy in therapeutics—Dr. W. K. Wills, 39.
Refraction, Golden rules of—Dr. E. E. Maddox (R.), 170.
Refraction of the eye—Dr. K. Campbell (R.), 71.
Reinhardt and D. Thomson, Drs. C. —A handbook of the open-air treatment and life in an open-air sanatorium (R.), 172.
Renal decapsulation, 62.
Rheumatism, The relationship of chorea and—Dr. J. J. S. Lucas, 239.
Rheumatism, The throat as a source of systemic infection in acute— Dr. P. W. Williams, 215.
Riddell, Dr. J. S.—A manual of ambulance (R.), 267.
Robinson, M.—A guide to urine testing (R.), 157.
Robson, A. W. M.—Diseases of the gall-bladder and bile-ducts (R.), 159.

Röntgen rays in dermatology, 343.
Röntgen rays in therapeutics and diagnosis—Dr. W. A. Pusey and E. W. Caldwell (R.), 71.
Royal national pension fund for nurses, 77, 96.

St. John ambulance brigade, 78.
Searle, R. B.—Medical tuberculosis (R.), 355.
Senator and S. Kaminer, Drs. H. [Eds.] —Health and disease in relation to marriage and the married state (R.), 347.
Serum therapy—Dr. R. T. Hewlett (R.), 167.
Shaw, Dr. J.—The physiognomy of mental diseases and degeneracy (R.), 263.
Sheild, Dr. A. M.—Surgical lectures and essays (R.), 352.
Sick, Preparations for the, 81, 181, 276, 366.
Skin, Pathology of the—Dr. J. M. H. Macleod (R.), 349.
Smith, E. N.—The management of lateral curvature of the spine (R.), 360.
Smith, W. J.—Surgical bandaging and dressings (R.), 72.
Smithsonian Institution, Report of the (R.), 171.
Sobotta, Dr. J.—Atlas and epitome of human histology and microscopic anatomy (R.), 68.
Society, Bristol Medico-Chirurgical, 87, 188, 377.
Spermatic cord, Torsion of the—Dr. J. L. Firth, 320.
Spinal cocainisation, Surgical analgesia by—W. J. Greer, 235.
Spinal injections of cocaine, Anæsthesia by intra-—Dr. A. S. Gubb, 231.
Spine, Lateral curvature of the—E. N. Smith (R.), 360.
Stephenson, Dr. S.—Ophthalmic nursing (R.), 170.
Sterilisation, Lessons in disinfection and—Dr. F. W. Andrewes (R.), 170.
Sterilisation of the hands—Dr. C. Leedham-Green (R.), 351.
Sterilisation of urethral instruments —Dr. H. T. Herring (R.), 160.
Stevens, Dr. A. A.—Modern materia medica and therapeutics (R.), 73.
Sturge, Miss E.—The misuse of medical charities, 273.
Subungual exostosis—W. R. Williams, 17.
Surgery, Manual of—Drs. A. Thomson and A. Miles (R.), 148, 358.
Surgery, Operative—Sir F. Treves (R.), 149; Dr. H. J. Waring (R.), 355.
Surgery, Reports on—T. Carwardine, 253; Dr. J. L. Firth, 62; C. A. Morton, 136, 255; Dr. J. Swain, 339.
Surgical diagnosis, Elements of—A. P. Gould (R.), 158.
Surgical lectures and essays—Dr. A. M. Sheild (R.), 352.
Surgical treatment, A manual of—Drs. W. W. Cheyne and F. F. Burghard (R.), 70.
Sutherland, Dr. W. G.—Dispensing made easy (R.), 163.
Swain, Dr. J.—Report on surgery, 339; The indications for operation in myo-fibroma of the uterus, 220; The varieties of abdominal tuberculosis: a clinical study, 31.
Swanzy, Dr. H. R.—A handbook of diseases of the eye and their treatment (R.), 169.
Swayne, Dr. W. C.—A case of Cæsarean section for obstruction of labour by a pelvic tumour, 120; Report on obstetrics, 140.
Symes, Dr. J. O.—A case of æstivo-autumnal malarial fever, with parasites of an unusual type, 126.
Syphilis, Clinical studies in—A. H. Ward (R.), 358.

Testis, The imperfectly descended —W. McA. Eccles (R.), 160.
Therapist, The pocket—Dr. T. S. Dowse (R.), 167.
Thomson and A. Miles, Drs. A.—Manual of surgery (R.), 148, 358.
Thomson, Drs. C. Reinhardt and D. —A handbook of the open-air treatment and life in an open-air sanatorium (R.), 172.
Throat as a source of systemic infection in acute rheumatism, On the—Dr. P. W. Williams, 215.
Tonkin, Dr. W. D. Neish and T. J. —Leprosy in Jamaica, 1.

Treves, Sir F.—A manual of operative surgery (R.), 149.
Tubal gestation, Case of—Dr. E. W. H. Groves, 46.
Tubby and R. Jones, A. H.—Modern methods in the surgery of paralyses (R.), 72.
Tuberculosis, Medical—R. B. Searle (R.), 355.
Tuberculous disease of the hip-joint in childhood and youth—C. A. Morton, 207.
Tunstall, Drs. F. J. Warwick and A. C.—"First aid" to the injured and sick (R.), 171.
Typhoid, Complications in, 131.

University College athletic ground, 77.
University College, Bristol, Recent honours obtained by students of, 77. Pass lists, 96, 191, 288, 382.
University College Colston Society, 74.
Urethral instruments, Sterilisation of—Dr. H. T. Herring (R.), 160.
Urine testing, A guide to—M. Robinson (R.), 157.
Urine, The examination of the—Dr. J. K. Watson (R.), 174.
Uterus, Cancer of the—Dr. A. H. N. Lewers (R.), 265.
Uterus, The indications for operation in myo-fibroma of the—Dr. J. Swain, 220.

Wallace, Dr. J. S.—The physiology of mastication and kindred studies (R.), 155.
Ward, A. H.—Clinical studies in syphilis (R.), 358.
Waring, Dr. H. J.—Manual of operative surgery (R.), 355.
Warwick and A. C. Tunstall, Drs. F. J.—"First aid" to the injured and sick (R.), 171.
Watson, Dr. J. K.—The examination of the urine (R.), 174.
White, Dr. W. H.—Forms of joint disease met with in general practice, 97.
Whiteford, C. H.—Ethyl chloride as a general anæsthetic, 49.
Whitelocke, R. H. A.—Football injuries (R.), 161.
Williams, Dr. E. C.—Notes on a case of blackwater fever, 128; Notes on a case of infantilism, 247.
Williams, Dr. P. W.—On the throat as a source of systemic infection in acute rheumatism, 215.
Williams, W. R.—Subungual exostosis, 17.
Willoughby, Dr. E. F.—Milk: its production and uses (R.), 153.
Wills, Dr. W. K.—Report on dermatology, 342; The present position of radiotherapy in therapeutics, 39.
Wingrave, Dr. W.—Adenoids (R.), 359.
Winsley sanatorium, 79, 180, 192, 365, 382.
Women, Diseases of—Dr. G. E. Herman (R.), 259; Dr. A. H. N. Lewers (R.), 172.
Women, Manual treatment of diseases of—Dr. G. Norström, (R.), 265.

X-rays, *see* Röntgen rays.

Yeo, Dr. I. B.—The therapeutics of mineral springs and climates (R.), 162.
Younger, E. G.—Insanity in everyday practice (R.), 357.

J. W. Arrowsmith, Printer, Quay Street, Bristol.

